# Circulating irisin levels in heart failure with preserved or reduced ejection fraction: A pilot study

**DOI:** 10.1371/journal.pone.0210320

**Published:** 2019-01-18

**Authors:** Andrea Silvestrini, Carmine Bruno, Edoardo Vergani, Angela Venuti, Angela Maria Rita Favuzzi, Francesco Guidi, Nicola Nicolotti, Elisabetta Meucci, Alvaro Mordente, Antonio Mancini

**Affiliations:** 1 Institute of Biochemistry and Clinical Biochemistry, Fondazione Policlinico Universitario A. Gemelli IRCCS, Roma—Università Cattolica del Sacro Cuore, Roma, Italy; 2 Internal Medicine Department, Division of Endocrinology, Fondazione Policlinico Universitario A. Gemelli IRCCS, Roma—Università Cattolica del Sacro Cuore, Roma, Italy; 3 Internal Medicine Department, Division of Internal Medicine and Cardiovascular Diseases, Fondazione Policlinico Universitario A. Gemelli IRCCS, Roma—Università Cattolica del Sacro, Roma, Italy; 4 Institute of General Pathology, Fondazione Policlinico Universitario A. Gemelli IRCCS, Roma—Università Cattolica del Sacro Cuore, Roma, Italy; 5 Hospital Medical Direction, Fondazione Policlinico Universitario A. Gemelli IRCCS, Roma—Università Cattolica del Sacro Cuore, Roma, Italy; Scuola Superiore Sant'Anna, ITALY

## Abstract

Irisin, a recently discovered myokine, has been considered a prognostic factor in several cardiovascular diseases. Nevertheless, no data are available on the role of irisin in patients with heart failure (HF), both with preserved (HFpEF) or reduced (HFrEF) ejection fraction. We have therefore evaluated the circulating irisin levels in HFpEF and HFrEF patients, correlating them with metabolic parameters and total antioxidant capacity (TAC), as index of oxidative stress. Irisin was significantly higher in HFpEF than in HFrEF patients (7.72 ± 0.76 vs 2.77 ± 0.77 ng/ml, respectively). An inverse correlation between irisin and TAC was found in HFpEF, but not in HFrEF. Conversely, no correlation was present with HOMA index. These data support the hypothesis that a different pathophysiological mechanism is involved in the two HF subtypes, and oxidative stress modulates irisin secretion.

## Introduction

Heart and skeletal muscle have emerged as endocrine organs due to the secretion of peptide hormones known as myokines [1]. Irisin is a novel myokine potentially capable of mimicking some of the most important metabolic and health-promoting benefits of exercise [2, 3], such as enhancing energy expenditure and reducing body weight, improving glucose homeostasis and insulin sensitivity, preventing or mitigating oxidative stress and systemic inflammatory state.

Irisin is a peptide hormone produced by a proteolytic cleavage of fibronectin type III domain-containing 5 (FNDC5), a transmembrane protein whose expression is induced by peroxisome proliferator-activated receptor (PPAR)-γ co-activator 1α (PGC-1α) [2] in response to exercise [2, 3] and/or oxidative stress [4].

In human, however, FNDC5 is highly expressed in cardiac muscle that produces more irisin than skeletal muscle [5]. Although human studies have suggested a tight association between circulating irisin levels and several cardiovascular diseases, nevertheless the physiological role of irisin in cardiomyocytes still remains unknown and controversial [1].

Heart failure (HF), which affects over 23 million people worldwide [6], is currently defined as “a complex clinical syndrome which results from any structural or functional impairment of ventricular filling or ejection of blood” [7]. The American College of Cardiology Foundation/American Heart Association guidelines have classified HF into two categories: (a) HF with reduced (≤ 40%) ejection fractions (HFrEF) also reported as systolic HF and (b) HF with preserved (≥ 50%) ejection fractions (HFpEF) also referred as diastolic HF [7]. HFrEF and HFpEF are two separate entities that differ considerably in aetiology, pathophysiology, clinical characteristics, and therapeutic strategies [8, 9, 10].

Therefore, in view of the putative role of irisin in prevention, control and therapy of numerous metabolic disorders [11,12] implied in HF, we aimed to evaluate the circulating irisin levels in HFrEF and HFpEF patients and correlate them with several metabolic and oxidative parameters.

## Materials and methods

Subjects involved in this study were admitted to the University Hospital “Fondazione Policlinico Universitario A. Gemelli IRCCS” Dept. of Internal Medicine and the study was conducted in accordance with the declaration of Helsinki, as revised in 2013. The study protocol was approved by our centre’s ethics committee (School of Medicine, Catholic University) and written informed consent was obtained from all patients. Two senior cardiologists separately confirmed the diagnosis of HF based on clinical history, physical examination, laboratory and echocardiographic parameters, according to the European Society of Cardiology Guidelines for the Management of Heart Failure [13]. To meet HFrEF inclusion criteria, patients had to present clinical symptoms and signs of HF with an EF < 40%. Conversely, HFpEF patients, together with clinical symptoms and signs of HF, had to present an EF at least of 50% with an NT-proBNP > 123 pg/ml and at least one additional criterion that included: a) relevant structural heart disease (left ventricle hypertrophy and/or left atrial enlargement); b) diastolic dysfunction.

Participants were excluded if they had uncontrolled hypertension (blood pressure > 140 mmHg/90 mmHg), alcoholism, drug abuse, abnormal hepatic function (transaminases > twice the upper limit of normal), end stage renal disease, malabsorption syndromes, gastro-esophageal reflux disease. Fifty-two subjects were assessed for eligibility; 5 refused to participate and 7 not meeting inclusion criteria. Thus, we included a total of forty patients in our study. Eighteen patients with HFrEF (15 males), aged 42–88 years (mean 69.2) and twenty-two patients with HFpEF (16 males), aged 64–88 years (mean 75.8), were recruited. All of them were Caucasian; they were treated by conventional therapy according to ESC guidelines (betablockers n = 14 HFpEF and n = 16 HFrEF; ACE-inhibitors n = 8 HFpEF and n = 7 HFrEF; angiotensin receptor blockade n = 6 HFpEF and n = 7 HFrEF; diuretics n = 9 HFpEF and n = 17 HFrEF; Ivabradin n = 1 HFpEF and n = 1 HFrEF). Comorbidities, as expected, were more prevalent in HFpEF patients (41% T2DM, 72% hypertension, 36% atrial fibrillation, 68% peripheral atherosclerosis, 63% non-end stage chronic kidney disease, 36% COPD) than in HFrEF patients (33% T2DM, 39% hypertension, 44% atrial fibrillation, 6% peripheral atherosclerosis, 33% non-end stage chronic kidney disease, 16% COPD). The two groups were not significantly different for age, sex, body mass, NYHA classification (all belonged to class II or III) and levels of physical activity (which was confined to sedentary activity).

Between 08.30 and 09.00 a.m., after an overnight fasting, blood samples were collected in a 6 ml vacutainer tube containing lithium heparin and immediately centrifuged (3000× g for 15 min at 4°C). The obtained plasma were collected and stored at -80°C until assayed. Fasting glucose and insulin levels were quantified with commercial kits using ADVIA 2400 automatic analyser (Siemens, Italy). Serum concentrations of N-terminal pro-B-type natriuretic peptide (NT-proBNP) were measured by an electrochemiluminescence immunoassays on a Roche modular E170 analyser (Roche diagnostic; Indianapolis, USA). Total antioxidant capacity (TAC) was evaluated with the method of Rice-Evans [14], modified in our laboratory as previously reported [15]. The method is based on the interaction between the system H_2_O_2_-metmyoglobin with the chromogen ABTS, whose radical form is spectrophotometrically detectable. The latency time (LAG phase in sec) before the appearance of radical species is proportional to the antioxidant concentration in the sample. Coefficient of variation (CV) for intra-assay CV (%) and inter-assay CV (%) variations were 0.54–1.59 and 3.6–6.1, respectively.

Circulating irisin levels have been quantified on plasma samples by a specific competitive enzyme immunoassay kit (Cat. No. EK-067-029 from Phoenix Pharmaceuticals, Karlsruhe, Germany) which has been previously validated by mass spectrometry analysis [16]. The intra- and inter-assay variations were less than 10% and 15%, respectively and the detection limit was 0.1 ng/ml. Optical density at 450 nm was measured, with a reading time of 1 sec, using a microtiter plate reader (Victor3; Perkin Elmer, USA) with precision at 450 nm < 0.5% and temperature control set at 25°C. Analyses were performed in duplicate.

The homeostatic model assessment (HOMA-IR) was used as an index of insulin resistance and was obtained from the fasting blood insulin (immunoreactive insulin: IRI, μUI/mL) concentration and the fasting blood sugar (FBS, mg/dl) level early in the morning, based on the equation: HOMA-IR = (IRI×FBS)/405.

Two-dimensional echocardiographic evaluation was performed (Echocardiography Philips, Affiniti 70C), measuring parameters described in Table 2.

The Mann-Whitney U test was employed to evaluate differences between the two groups of subjects. A p value ≤ 0.05 was considered statistically significant. Linear regression and non-linear (semilogarithmic) analysis was employed to correlate irisin with biochemical and echocardiographic parameters.

A multiple logistic regression model was developed to quantify the association between HFpEF (HFrEF = 0) and irisin levels. The only covariates included in the model were irisin levels and NT-proBNP (due to numbers of observations in the final model). The relationship between HFpEF and irisin levels has been reported as Odds Ratios (ORs) and 95% confidence intervals (CIs). The goodness of fit of the final model was assessed using the Hosmer-Lemeshow test [17]. Descriptive, univariate and multivariate analyses were performed with STATA version 11.0.

## Results and discussion

Baseline characteristics (number of patients, gender, NYHA class, age, BMI, NT-proBNP, HOMA-IR and TAC) of the patients with HFrEF and HFpEF are summarized in Table 1. Among HFpEF, 9 out of 22 patients were affected by diabetes and were not included in the calculation of HOMA-IR index. Similarly, 5 out of 18 HFrEF patients were excluded for HOMA-IR calculation. There was no significant difference between the two groups except for NT-proBNP, that was significantly higher in HFrEF than in HFpEF patients thus confirming what already reported in numerous reports (see [18] and references therein). Echocardiographic parameters of HF patients were reported in Table 2. As shown, the ejection fraction is, by definition, significantly different between the two groups. Other significant differences were found in LVEDD, LVESD, LVEDV, LVESV all higher in HFrEF, as a distinctive feature of this disease.

**Table 1:** Baseline characteristics of patients with heart failure with reduced (HFrEF) and preserved (HFpEF) ejection fraction. Data are presented as mean ± standard error of the mean (SEM).

	HFrEF	HFpEF
Number of patients	18 (15 males)	22 (14 males)
Age	69.2 ± 2.8	75.7 ± 1.8
NYHA class	II (n = 9) III (n = 9)	II (n = 16) III (n = 6)
BMI (Kg/m^2^)	26.54 ± 0.95	28.9 ± 1.30
NT-proBNP (pg/ml)	6000.07 ± 2297.22	2548.40 ± 551.11*
HOMA-IR	2.30 ± 0.38	2.73 ± 0.54
TAC (sec)	68.7 ± 4.7	75.8 ± 7.5

*p < 0.05

**Table 2:** Echocardiographic parameters of HFrEF and HFpEF patients. Data are presented as mean ± standard error of the mean (SEM).

	HFrEF	HFpEF
^1^LVEDD (mm)	64.87±6.28	49.17±1.32*
LVESD (mm)	41.37±1.28	31.94±1.31*
LVEDV (ml)	140.9±9.28	98.78±5.14*
LVESV (ml)	84.1±10.4	43.85±2.35*
IVS (mm)	12.83±2.42	12.84±0.36
PW (mm)	11.5±0.86	10.3±0.41
E (mm/s)	605.4±169.3	610.62±86.35
A (mm/s)	596.25±181.8	713.69±83.3
Dt (ms)	212±40.5	219.25±18.95
EF %	36.7±2.7	56.7±1.3*
E/E’	10.3±1.5	12.1±0.84
E/A	0.79±0.12	1.15±0.36
LAV (ml)	86±9.16	82.68±4.95
LAVI (ml/m^2^)	n/a	44.56±2.55
TPV (m/s)	n/a	2.74±0.08
TAPSE (mm)	19.73±1.25	22.4±0.84
SPAP (mmHg)	36.54±2.24	35.79±2.64

^**1**^Left ventricular end-diastolic diameter (LVEDD), left ventricular end-systolic diameter (LVESD), left ventricular end-diastolic volume (LVEDV), left ventricular end-systolic volume (LVESV), septal thickness (IVS), posterior wall thickness (PW), peak E-wave velocity (E), peak A-wave velocity (A), deceleration time (Dt), ejection fraction (EF %), pulsed-wave TDI E’ velocity (E’), E/E’ ratio, E/A ratio, left atrial volume (LAV), indexed left atrial volume (LAVI), tricuspidal peak velocity (TPV), tricuspid annular plane systolic excursion (TAPSE), and systolic pulmonary artery pressure (SPAP).

*p < 0.05

**Fig 1:**
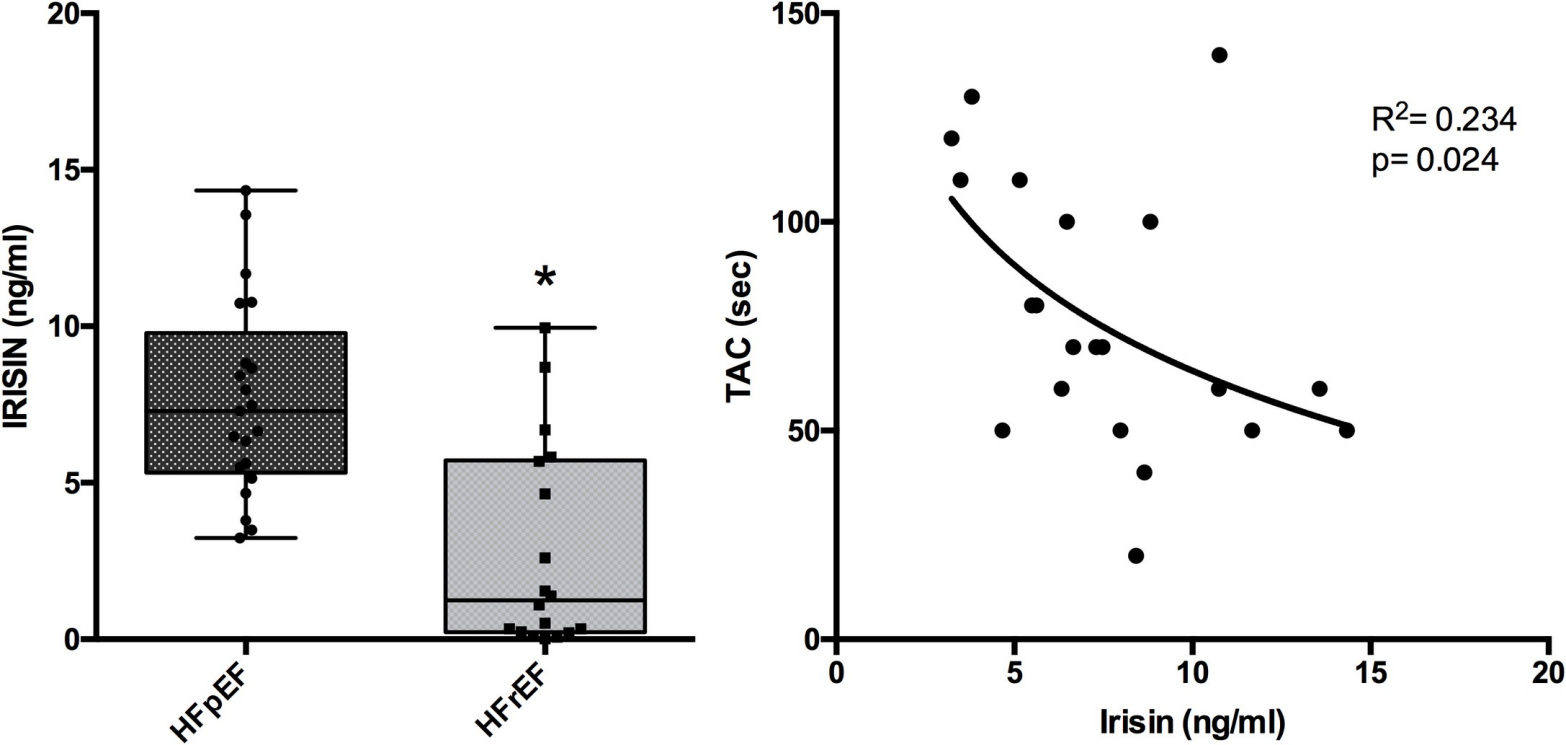
Irisin levels in patients with heart failure with reduced (HFrEF) and preserved (HFpEF) ejection fraction. Left panel shows a Box-Plot comparing irisin levels in HFpEF with that in HFrEF (* p < 0.05). Right panel shows the correlation between irisin levels and TAC (in sec) in HFpEF.

As shown in Fig 1 (left panel), circulating irisin levels in HFpEF were significantly higher (7.72 ± 0.76 ng/ml) than in HFrEF patients (2.77 ± 0.77 ng/ml). Moreover, a logarithmic correlation between TAC and irisin parameters was found in HFpEF patients (R^2^ = 0,234 and p = 0,024) (Fig 1, right panel). Conversely, in HFrEF patients, irisin levels and TAC did not significantly correlate both in linear and in logarithmic regression analysis.

Finally, no significant correlation was found between irisin and BMI or HOMA-IR for both HFrEF and HFpEF groups. Moreover, in both groups of patients, there was no significant correlation between irisin and each of the echocardiographic parameters reported in Table 2.

At the logistic regression, only irisin levels show a significant association with HFpEF (OR = 1.76; 95% CI: 1.18–2.61; p < 0.01). The Hosmer-Lemeshow test supports the goodness of fit of the final model (p = 0.83).

To the best of our knowledge, this is the first study comparing irisin levels between HFpEF and HFrEF patients that shows higher irisin levels in HFpEF, despite a higher average age of this group.

The increased levels of irisin observed in HFpEF patients might be due, rather than a passive release, to an enhanced secretion aimed to compensate for the development of a putative “irisin resistance” [12] and to maximize the beneficial effects of irisin on metabolic comorbidities as well as on endothelium dysfunction.

Irisin, indeed, in addition to regulating energy metabolism, improves endothelial function [19, 20] by its anti-inflammatory and anti-oxidizing properties.

Moreover, it has been suggested that blood levels of irisin may be regulated by oxidative stress that increases irisin secretion whereas antioxidants decrease it [4]. Our findings, confirming this suggestion, show an inverse correlation between irisin and TAC in HFpEF patients.

Nevertheless, there are some potential limitations of the present study as expected by a pilot study. Firstly, the number of subjects in both groups is slightly small, so its statistical power is limited, and our findings will need to be confirmed in a larger population. Secondly, this pilot study and the power analysis cannot draw a cause-effect conclusion about irisin/TAC correlation in heart diseases patients. Further studies are needed to explain the physiological role of irisin in myocardium and its correlation to HF. Finally, we cannot discriminate the source of circulating irisin (skeletal muscle vs heart myocytes) in such patients that remain a pilot study.

In conclusion, this pilot study represent the first observation about irisin in different models of HF. Moreover, despite these limitations, the present preliminary data are in favour of the concept that a different pathogenetic model is involved in the two HF subclasses and suggest that irisin levels in HFpEF could be an index of multi-systemic disease, according to the recent paradigm for HF [10], rather than a primitive heart disease.
